# What Do Mismatch Negativity (MMN) Responses Tell Us About Tinnitus?

**DOI:** 10.1007/s10162-024-00970-1

**Published:** 2024-12-16

**Authors:** Ekaterina A. Yukhnovich, Kai Alter, William Sedley

**Affiliations:** 1 Newcastle University Medical School, Newcastle Upon Tyne, NE2 4HH UK; 2 Faculty of Modern and Medieval Languages and Linguistics and the Languages Sciences Interdisciplinary Research Centre, University of Cambridge, Cambridge, UK

**Keywords:** Tinnitus, Mismatch negativity, Predictive coding, Biomarker, MMN, Review, Tinnitus mechanisms

## Abstract

Due to the heterogeneous causes, symptoms and associated comorbidities with tinnitus, there remains an unmet need for a clear biomarker of tinnitus presence. Previous research has suggested a “final pathway” of tinnitus presence, which occurs regardless of the specific mechanisms that resulted in alterations of auditory predictions and, eventually, tinnitus perception. Predictive inference mechanisms have been proposed as the possible basis for this final unifying pathway. A commonly used measure of prediction violation is mismatch negativity (MMN), an electrical potential generated in response to most stimuli that violate an established regularity. This narrative review discusses 16 studies comparing MMN between tinnitus and non-tinnitus groups. Methods varied considerably, including type of deviant, type of paradigm and carrier frequency. A minority of studies matched groups for age, sex and hearing, with few measuring hyperacusis. Frequency deviants were the most widely studied; at frequencies remote from tinnitus, MMN was consistently smaller in tinnitus groups, though hyperacusis or altered distress or attention could not be ruled out as explanatory factors. Few studies have used tinnitus-related frequencies; these showed larger MMN to upward frequency deviants above the tinnitus frequency, and larger MMN to upward intensity deviants at or close to the tinnitus frequency. However, the latter appears a correlate of hyperacusis rather than tinnitus, and tinnitus groups without hyperacusis instead show larger MMN to downward intensity deviants than controls. Other factors that affect MMN amplitudes included age, attention, and the specific characteristics of the range of stimuli across a particular experiment paradigm. As such, MMN cannot presently be considered a specific biomarker of tinnitus, but showed potential to objectively characterise a number of auditory processing traits relevant to tinnitus and hyperacusis.

**Supplementary Information:**

The online version contains supplementary material available at 10.1007/s10162-024-00970-1.

## Introduction

### Mechanisms of Tinnitus

Tinnitus stems, at least partially, from functional changes at different levels of the auditory pathway [1, 2]. The classical explanation of tinnitus appearance is that peripheral auditory damage induces tinnitus through homeostatic plasticity processes acting to preserve mean firing rates in the face of diminished input, which in turn elicit increased spontaneous firing, excessive neural synchrony and/or tonotopic map reorganisation. Other possible tinnitus-causing factors are changes in inhibitory processes, for instance in thalamic nuclei, or facilitation of non-auditory input into the auditory pathway [1, 3]. Coordination between excitatory and inhibitory firing allows neural gain to adjust or “compensate” for any changes in its function by altering intrinsic excitability, receptor expression, neurotransmitter release volume or probability [2, 4]. There is additive noise (that can occur at multiple different levels) which possibly compensates for overt hearing loss. This central noise may be a part of homeostasis maintenance within the brain, in part through a bottom-up process of adaptive stochastic resonance (SR). SR theory states that addition of uncorrelated neural noise allows a particular narrow range of signals that are weak, for example, due to impaired cochlea neurons, to increase via SR and reach a threshold in the nonlinear auditory system [5]. These factors, however, may alter sensory perception [6].

Predictive coding is a common framework which explains perception as a result of constant updating of internal models of the environment based on sensory input. From the lower levels of the sensory system, we receive current sensory input. Higher level predictions about the sensory input are based on prior beliefs and expectations. The bottom-up sensory information ascends through the neural pathway via excitatory neurons, and top-down predictions are relayed down by inhibitory neurons, to be compared [7]. Any discrepancies between them generate a *prediction* *error* signal, which can then be used to update the model to bring its predictions more in line with sensory input. Prediction errors and predictions are each weighted by *precision*, which reflects the level of confidence, reliability or importance the system attaches to it [8]. Where prior predictions carry a lot of precision, the resulting *posterior* perceptual inference is closer to the prior prediction than the sensory input. On the other hand, when prior predictions carry relatively less precision, prediction errors have more influence over the resulting posterior perceptual inference, aligning perceptual inference more closely to the sensory input. Precision is principally encoded by postsynaptic gain, which at a cortical level is principally controlled by the basal forebrain cholinergic system [9–11]. According to this view, attention operates via (and is the same as) increasing the precision of specific topographic regions of sensory input.

In predictive coding frameworks, tinnitus is argued to occur when spontaneous activity in the auditory pathway is subject to sufficiently high precision to create an inferred percept by over-riding the default auditory prediction of silence [7]. After the tinnitus signal is recognised and learned, which can occur as a result of neural activity altered by any of the tinnitus onset mechanisms mentioned above, auditory predictions are adjusted accordingly, and a much lower level of sensory precision is required in the future to lead to the perception of tinnitus. Therefore, an individual becomes more predisposed to developing tinnitus, as weaker spontaneous signals could cross the threshold into conscious perception of tinnitus, or the person may more readily develop tinnitus from events such as noise exposure (that leads to heightened neural activity within the auditory system) or either chronic or even temporary hearing loss (such as ear plugging and infections).

While this view remains largely theoretical, it could potentially unite many contributory mechanisms to tinnitus into a common framework and highlight the importance of also considering how differences or alterations in auditory predictive coding processes might either predispose people to developing tinnitus and/or result from the presence of chronic tinnitus.

## What is Mismatch Negativity?

**Fig. 1:**
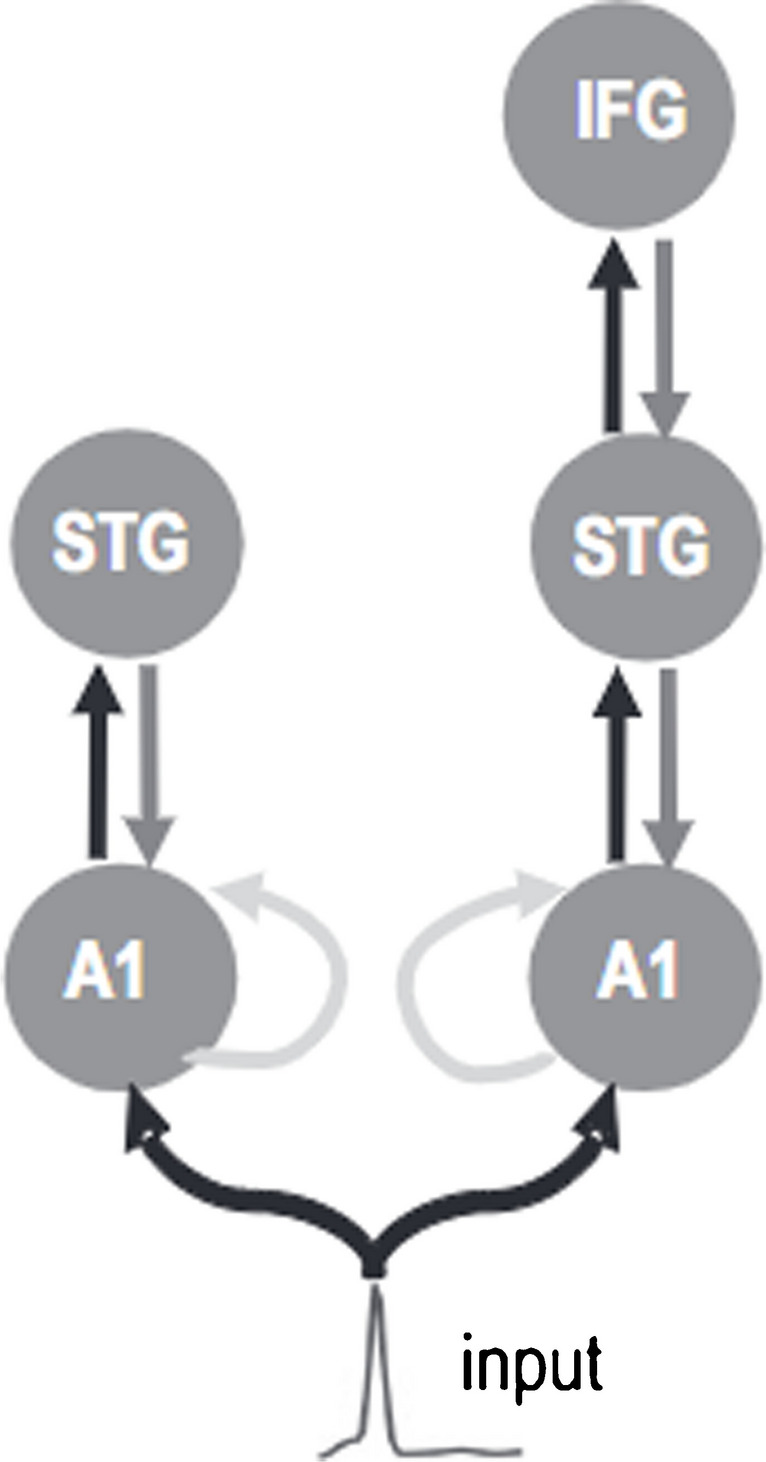
Winning dynamic causal model to explain auditory MMN (reproduced from [21]). A1 is the primary auditory cortex, STG is the supra-temporal gyrus and IFG is the inferior temporal gyrus. Black arrows represent forward connections, dark grey arrows show backward connections and light grey arrows show intrinsic connections. These connections can be altered by factors such as age and attention towards the stimuli

Neural correlates of prediction error signals are commonly measured using electroencephalography (EEG) [12]. Mismatch negativity (MMN) is a pre-attentive evoked potential that indicates sensory regularity violations in comparison to recent sensory context [13–15]. It quantitatively represents prediction errors created from these comparisons [16], and its magnitude also indicates the precision of the prediction being violated. While MMN does not require attention, it is increased by attention [17], which can be considered as it also quantitatively reflects the precision of the sensory input that violates the prediction. However, it is not a “pure” marker of prediction error, as it also reflects other processes such as adaptation [18]. Sensory memory traces may underlie MMN, as these grow in tandem with repetition of the regular sensory context, allowing for a stronger response during comparison between the new incoming information and repetitive recent events [19, 20]. In the auditory system, MMN generation has been attributed to changes in extrinsic and intrinsic connectivity in a network comprising bilateral primary auditory cortices and superior temporal gyri (secondary auditory cortex), prefrontal cortical regions, and the insula [16, 19, 20]. Studies that collected responses to frequency deviants through a number of paradigm types using MEG were fitted to a number of potential network connectivity models using dynamic causal modelling (DCM); the best model was one with forward and back connections between the primary auditory cortex (A1), superior temporal gyrus (STG) and the right inferior frontal gyrus (rIFG), as well as intrinsic connectivity within A1 that represents adaptation mechanisms (Fig. 1) [16, 21]. This common network was confirmed in other auditory deviant types as well, but with small differences between deviant types [22, 23]. Importantly, increasing age affects the connectivity underlying MMN through stronger inhibition of the rIFG but increased connectivity between STG and A1. This possibly occurs due to decreased ability to create a sensory memory trace [24]. Additionally, a DCM study showed that attentional modulation can increase the gain of intrinsic inhibitory neurons within A1 and inhibitory backward connections, which results in increased MMN amplitudes to attended compared to unattended stimuli [17]. Exposure to long-term occupational noise, even without explicit markers of hearing loss, may also alter the primary source of MMN: in participants who were exposed to noise, the maximum current strength appeared more lateralised towards the right temporal region whereas in the unexposed group, maximum current strength appeared more frontal (though these two groups also had significantly different attention scores on a cognitive test) [25]. Other factors, such as sex and psychological factors, can also alter latencies and amplitudes of the MMN response (e.g. [26–29]).

**Fig. 2:**
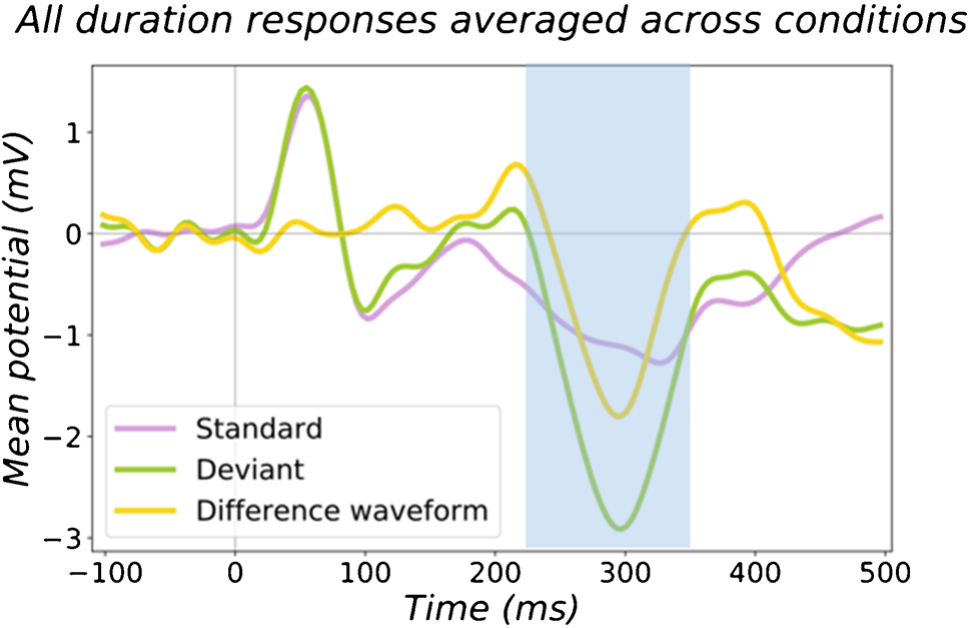
Example MMN and its calculation. (Reproduced from [35]) In this study, standards (pink) were isochronous 300* ms* pure tones of fixed frequency, and deviant tones (green) were shorter in duration at 150 ms. MMN is evident in the difference waveform (yellow), calculated by subtracting the standard response (purple) from the deviant response (green). The blue rectangle is for illustrative purposes only, to highlight the approximate timeframe of the MMN response in this example. The waveform shown was recorded by FCz channel, with averaged mastoids as the reference

Experimentally, MMN can be elicited by even small perceptual changes (deviants) to regular (standard) stimuli using auditory stimuli through oddball paradigms [13]. Within the auditory modality, a deviation could occur in simple sound features including frequency, intensity, duration, location or omission (silent gap). Amplitudes of auditory MMN responses have been shown to have good test–retest reliability over separate recordings when conducted on the same subjects on multiple occasions, or in multisite experiments, in a variety of paradigm types [30–32]. MMN is represented in EEG data as a negative polarity waveform over the vertex, typically using a mastoid reference. It is calculated by subtracting the responses to standard auditory events from the response to a deviant auditory event, and usually occurs around 150–250 ms after the onset of the deviant (Fig. 2) [1, 4], though the duration and latency can depend on the size of the deviant, with larger deviants giving MMN responses that begin earlier, are shorter in duration and larger in amplitude [33, 34]. Timeframes in which MMN is studied however may differ depending on the paradigm, as simpler deviations may create delayed and prolonged MMN waveforms even in healthy participants (e.g. Figure 2 shows responses to simple duration deviants, and has a later MMN timeframe) [33].

### Need for a Biomarker

Due to the heterogeneity of potential causes, symptoms and comorbidity, classification of tinnitus has been difficult [1]. Even much of the research into risk factors of tinnitus (except hearing loss-related factors) is not yet conclusive enough to determine causal relationships [36]. Furthermore, it is not clear which mechanisms are required for tinnitus development, or instead are correlates of closely related conditions such as hearing loss and hyperacusis [37]. Hearing loss has been established as the largest risk factor of tinnitus [38, 39]. Hyperacusis (a disorder in which normal environmental sounds are experienced as uncomfortably loud) also often co-occurs with tinnitus [40]. Frequently, these factors are not subject to stringent enough controls in tinnitus research to know which condition(s) actually relate to any observed neural changes. In this review, we discuss whether MMN can be used to indicate tinnitus presence in all participants, whether or not they have these confounding conditions.

Another difficulty in determining tinnitus mechanisms is that tinnitus research has been heavily guided by animal studies; there is no standardised animal model, and limited correlation between different putative measures of tinnitus in animals, such as the acoustic startle reflex and conditioned behaviour [1]. A reliable diagnostic test for tinnitus in animals that has also been validated in humans would allow tinnitus research findings from human and animal research to be better aligned.

An ideal tinnitus biomarker would be one that indicates the presence of tinnitus across all the potential subgroups, irrespective of specific contributory mechanisms. Such a biomarker would not only help to better understand essential core tinnitus mechanisms, but also allow treatment studies to more objectively determine their effectiveness across tinnitus groups. As explained above, predictive coding frameworks might potentially unite the different mechanisms that potentially lead to subjective tinnitus by providing a potential “final” measurement that occurs in all individuals with tinnitus regardless of initial causes [41]. It is, therefore, appealing to search for tinnitus biomarkers that reveal aspects of auditory predictive processing that are common to a majority of, or even all, cases of tinnitus. However, the question remains as to whether MMN can act as such as biomarker.

## MMN and Tinnitus

Using studies that include deviations in simple sound features, one might identify changes in MMN associated with tinnitus in two possible ways: the first is that the presence of tinnitus causes changes in MMN as a result of either the sound properties itself, altered auditory predictions, or secondary to distress or altered attention; and the second possibility is that MMN could reflect altered sound processing that might affect one’s propensity to tinnitus (e.g. determination of regularities in auditory signals). These possibilities are not mutually exclusive. A number of studies have been carried out to investigate alterations in deviance detection in chronic subjective tinnitus compared to controls, using different types of oddball paradigm (Tables 1 and 2). Studies that did not feature a control group were not included because tinnitus mechanisms and biomarkers were of particular interest, rather than correlates of tinnitus distress.

**Table 1:**
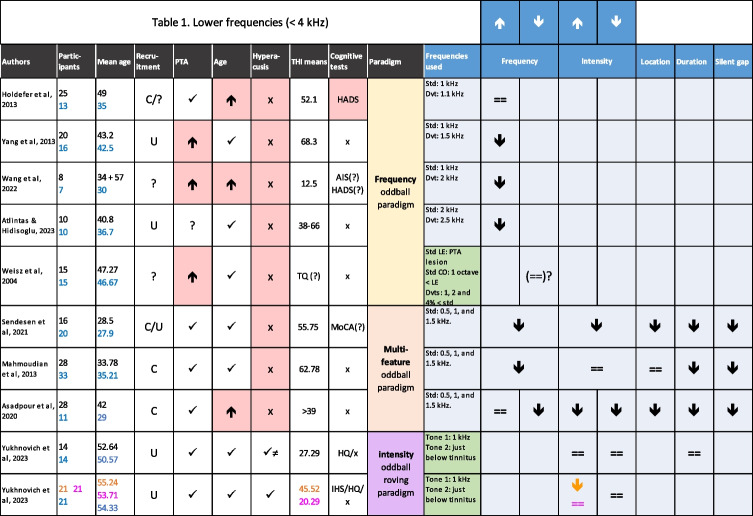
Published MMN studies on tinnitus that used stimuli frequencies < 4 kHz that included a control group

Green cells in the ‘Frequencies used’ column indicate studies also using stimuli with frequencies above 4 kHz (i.e. studies that are also listed in Table 2), as well as lower frequencies. In columns named “Participants” and “Mean age”, numbers for tinnitus samples are shown in black; numbers for controls are shown in blue (). In the “Recruitment” column, C = clinic, U = university, ? = unknown. In “PTA” and “Age” columns, ⬆ = higher value or stronger amplitude in the tinnitus sample compared to controls; ⬇ = lower value or lower amplitude. In the “Hyperacusis” column, (✓) = hyperacusis excluded, ≠ sign means values were significantly different. In the “Cognitive tests” column, HADS = hospital anxiety and depression scale (>8 = anxiety, >9 = depression on subsections), AIS = Athens Insomnia Scale, MoCA = Montreal Cognitive Assessment Test. More information in Supplementary materials. In the last row [], orange = tinnitus with hyperacusis (T+H+), purple = tinnitus without hyperacusis (T+H-)

**Table 2:**
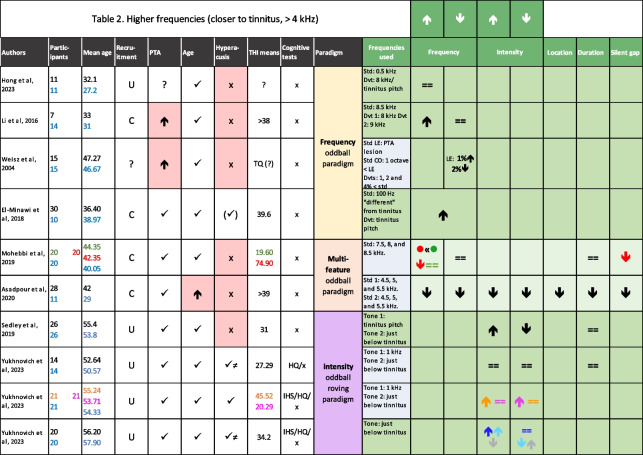
Published MMN studies on tinnitus that used stimuli frequencies > 4 kHzthat included a control group

Published MMN studies on tinnitus that used stimuli frequencies > 4 kHzthat included a control group. Blue cells in the “Frequencies used” column indicate studies in which stimuli included tones of frequencies below 4 kHz (i.e. studies that are also listed in Table 1. Darker-green cells represent studies that used stimuli frequencies that were based on tinnitus pitch match. In row 5 (Mohebbi et al., 2019), green and red numbers/symbols indicate participants with non-bothersome and bothersome tinnitus, respectively. In the second-to-last row [], orange = tinnitus with hyperacusis (T + H +), purple = tinnitus without hyperacusis (T + H-). In the last row [], dark blue = auditory task, light blue = neutral task, grey = visual task. In “Participants” and “Mean age” columns, numbers for tinnitus samples are shown in black; numbers for controls are shown in blue. In the “Recruitment” column, C = clinic, U = university, ? = unknown. In “PTA” and “Age” columns, ⬆= higher value or stronger amplitude in the tinnitus sample compared to controls; ⬇= lower value or lower amplitude. In the “Hyperacusis” column, (✓) = hyperacusis excluded, ≠ sign means values were significantly different. In the “Cognitive tests” column, HADS = hospital anxiety and depression scale (> 8 = anxiety, > 9 = depression on subsections), AIS = Athens Insomnia Scale, MoCA = Montreal Cognitive Assessment Test. More information in Supplementary materials

There are some confounds that have affected MMN research generally, as well as tinnitus research specifically, including age, hearing and sex differences as well as comorbid hyperacusis (Tables 1 and 2). There remains a need to disentangle the effects of each factor on MMN responses, in order to be able to interpret which of the observed changes truly relate to tinnitus directly, and what these changes signify about the mechanisms of tinnitus and/or the tinnitus status of the individual(s).

### Types of MMN (oddball) Paradigm Used in Tinnitus Research

**Fig. 3:**
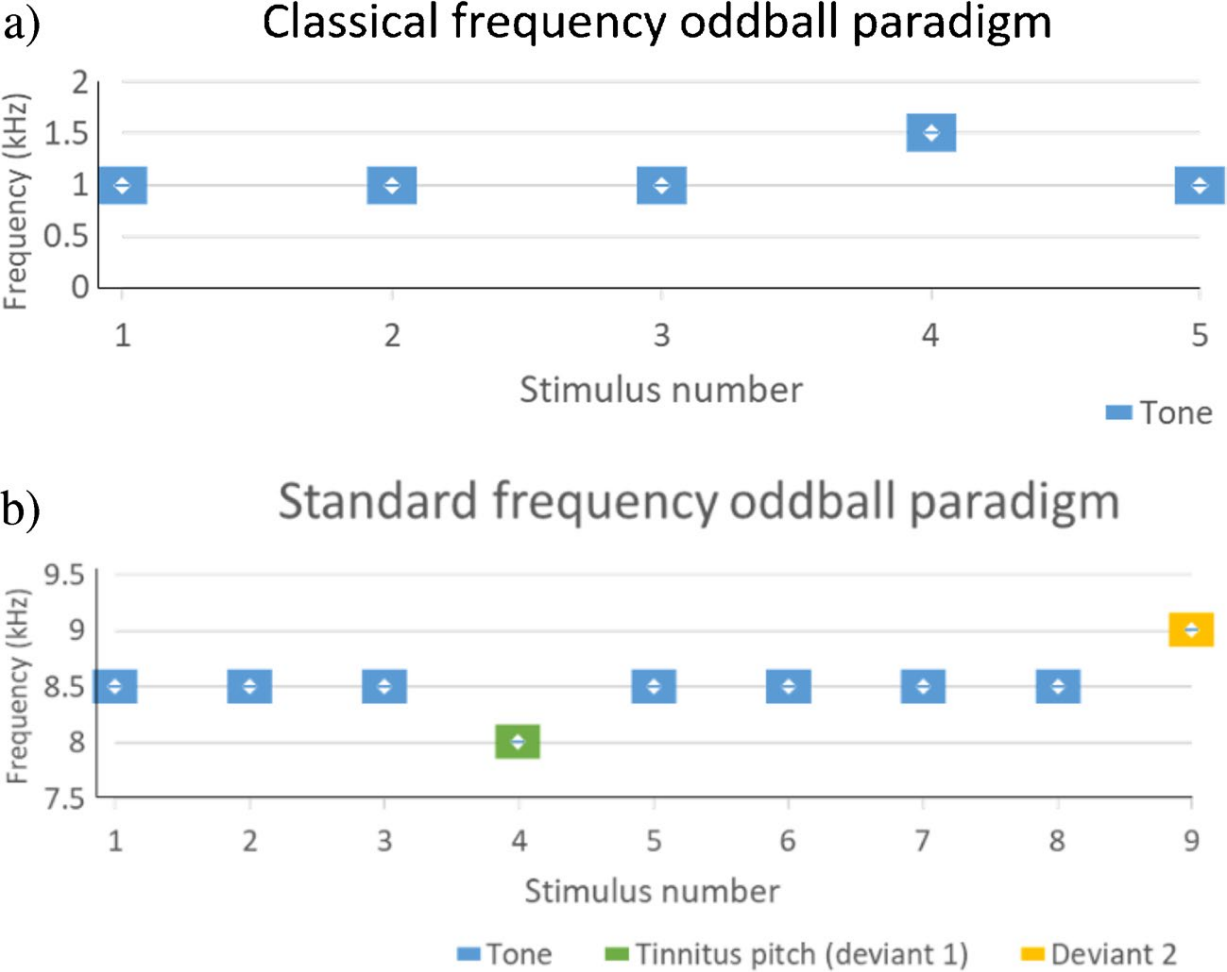
Examples of classical oddball paradigms. **a** A classicalfrequency paradigm with tones at 1 kHz. The rare 1.5 kHz deviant tone is displayed as the 4.th stimulus. **b** Another type ofclassical oddball paradigm that has been used in tinnitus research, where one of the tones is matched to the tinnitus pitch of a participant. The standard tones are usually slightly different frequency to tinnitus (e.g. in this example they are set 0.5 kHz higher than the tinnitus pitch). These paradigms can also have a deviant in the opposite direction to the direction of the tinnitus pitch deviant (in this example, the tinnitus pitch is a downward deviant and the second deviant is an upward deviant)

**Fig. 4:**
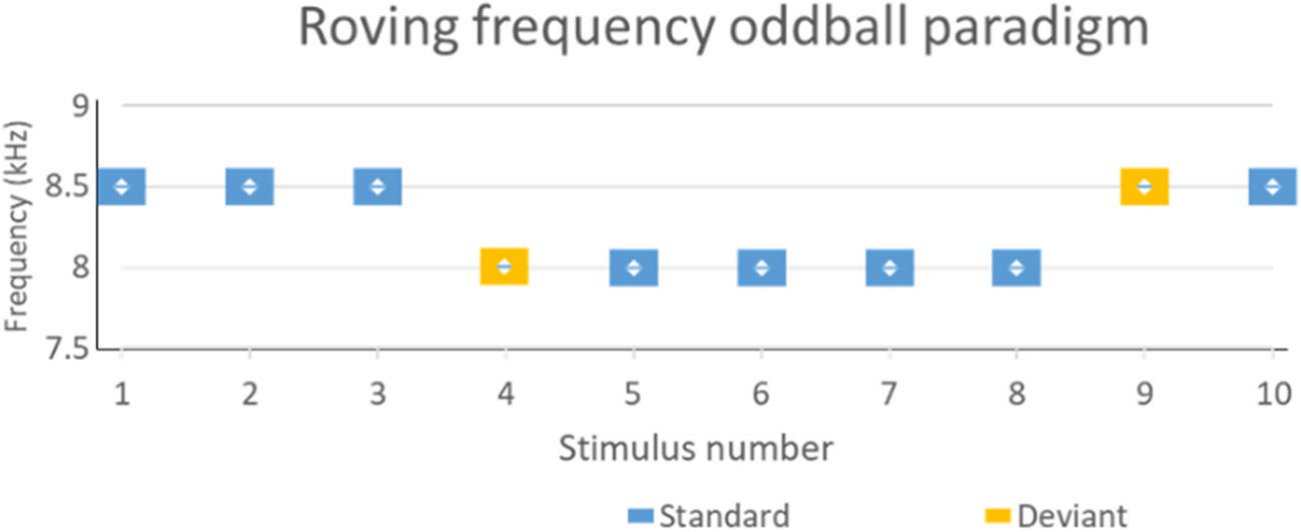
Example of a roving oddball paradigm. In this example, the frequency of the tones is the roving factor. The first tone at each frequency is the deviant, but this frequency becomes the new standard once it is repeated

**Fig. 5:**
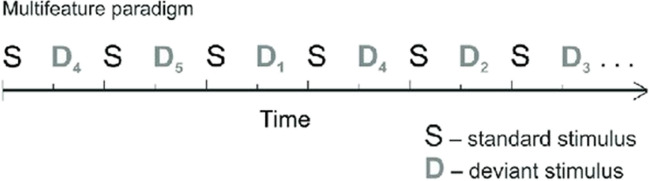
Example of a multi-feature oddball paradigm. (Reproduced from [44]) The size of the tone marker represents the intensity (typically, 2nd and 3rd tone being 3 and 6 dB lower than the 1st, respectively). Every second stimulus is a deviantin one factor, but has been preceded by numerous consecutive “standards” with respect to that factor

In the classical oddball paradigm, a standard stimulus is repeated, with an unexpected stimulus (deviating in a particular sound feature), randomly replacing up to approximately 20% of stimuli (Fig. 3) (e.g. [42].). Typically, a minimum number of consecutive standards are required prior to each deviant. Other types of oddball paradigm include the roving paradigm, in which there are two types of standard stimuli, and deviants are the pseudo-random transitions from one standard tone to the other (Fig. 4) (e.g. [43]). Another type of oddball paradigm is a multi-feature paradigm (Fig. 5) (e.g. [33]). Every second stimulus is a deviant of some kind (interspersed with standards), but each deviant only differs in one of the following characteristics: frequency (usually ± 10%), intensity (usually ± 10 dB), duration (25 ms rather than 75 ms), perceived location (800 us latency difference between left and right stimuli sources) or there may be a silent gap (7 ms silent gap in the middle of the 75 ms stimulus tone).

### Frequency Deviants

So far in MMN and tinnitus literature, frequency has been the most explored sound feature, which seems logical based on the nature of tinnitus, and a similar focus in the wider MMN literature. Notably, the majority of these oddball studies, whether classical or multi-feature, focused on lower frequencies (commonly 1000 Hz). While this follows MMN research into other phenomena such as ageing, cognitive function and even Parkinson’s disease, this choice of frequency to study tinnitus is in other respects counter-intuitive, considering tinnitus pitch tends to be fairly high frequency, likely due to its high comorbidity with sensorineural hearing loss [29, 45–48]. Here, we separately consider MMN findings at frequencies remote from, and close to, tinnitus frequencies, using 4 kHz as an arbitrary initial cut-off, but also sub-categorising higher frequency stimulus studies based on whether stimuli were matched to participants’ specific tinnitus frequencies.

#### Frequencies Below 4 kHz

At a lower frequency (usually 1 or 2 kHz standard), three studies found a weaker MMN response amplitude to *upward* frequency deviants using a classical oddball paradigm [49–51], while two other studies found a similar response between participants with tinnitus and controls both in classical and multi-feature paradigms [42, 52]. The aforementioned multi-feature study also saw a reduced MMN amplitude in tinnitus in response to a *downward* frequency deviant [52]. However, another study found no differences in response to downward deviants [53]. Unfortunately, [53] did not report the exact frequencies used in their control condition, only mentioning that the standard tones were one octave below the edge frequency of each tinnitus participant, with three downward deviant conditions (1, 2 or 4% lower than standard frequency). The edge frequency is calculated by finding the largest difference in hearing threshold between one tested frequency and the next (e.g. 2000 Hz and 4000 Hz in standard Pure Tone Audiometry). Finally, there were also two multi-feature studies that did not differentiate between the directionality of the frequency deviant, who also found an overall weaker amplitude to these deviants [33, 54].

Some of the studies had limitations in terms of samples sizes and control matching, either for age or in hearing thresholds. Previous studies found reduced MMN amplitudes, associated with increasing age, in response to both upward and downward frequency deviants (as well as duration, but not intensity, deviants) [47, 55–57]. Unfortunately, the two studies that formally matched on both age and hearing did not differentiate between upward and downward frequency deviants; therefore, it is unclear whether the directionality of the deviant played a role in this set of findings. However, the only two studies that found similar MMN amplitudes in response to *upward* frequency deviants between the two groups were matched in hearing thresholds but had tinnitus participants who were significantly older than controls. Other factors that may have influenced those findings include the higher anxiety score in the tinnitus group, which has previously been related to larger MMN amplitudes, and the additional presence of distant higher frequencies within the paradigm [53, 58]. This may be an important factor for the MMN responses, in control participants in particular, as previous studies have shown different patterns, in an intensity oddball paradigm run on healthy participants, simply on account of blocks of a different frequency being present elsewhere in the experiment, even though the current and recently preceding stimuli were identical [35, 41, 59, 60]). These differences could occur through adaptive processes to a particular environment due to mechanisms such as frequency-specific adaptation [61, 62].

#### Frequencies Above 4 kHz

Six studies investigated frequency deviant effects at higher frequencies, three of which matched the stimulus frequencies to tinnitus pitch of their participants [63–65].

##### Tinnitus Pitch-Matched Stimuli

Two of the studies with tinnitus pitch-matched stimuli had age-matched control samples, with the tinnitus group having THI scores > 38 [63, 64]. Another study had age-matched control participants but did not report THI scores [65]. The latter study had the largest difference between the standard (500 Hz) and deviant (tinnitus match or 8 kHz) frequencies. The authors found that during a passive paradigm (with no active auditory task but with a distractor, i.e. a silent movie), there were no differences in MMN amplitude in response to an *upward* frequency deviant *toward*s the tinnitus frequency between tinnitus and control groups [65]. In [64], tinnitus participants were recruited in a clinical setting, based on matching their tinnitus pitch to 8 kHz. Eight kilohertz was used as a *downward* deviant (D1) from the standard (8.5 kHz). There was also an upward deviant set at 9 kHz (D2). The control group did not show a significant MMN response to either of the deviants (though there was a trend towards a larger response for D1). The tinnitus group showed a significantly stronger MMN amplitude to D2 which was further *away* from their tinnitus frequency but not D1, which was the tinnitus pitch match [64]. The tested ear of the tinnitus group on average had some hearing loss (up to 60 dB at 8 kHz) while controls had normal hearing, which may have affected responses both to D1 and the standard. Additionally, the hearing thresholds in the tinnitus group could have been lower for 9 kHz than 8 kHz, thus leading to a combined upward intensity and upward frequency deviant. The second study that used matched tinnitus frequency as a deviant used a standard tone that was 100 Hz “different” from the matched tinnitus pitch [63]. They did not specify the direction in which the deviant tone changed, but this stimulus set-up was probably most like D1 in [64]. This study formally matched both age and hearing thresholds of their participant groups [63]. Here, MMN amplitudes were larger in the tinnitus group than the controls in response to the frequency change *towards* the tinnitus match, which was incongruent with the D1 finding in [64]. Differences between this and the other two studies might lie in better hearing matching or exclusion of participants with low pure-tone loudness discomfort level (LDL) scores [63]. LDLs have been associated with presence of hyperacusis, which remains a significant confounding factor in tinnitus research [37, 66–68]. However, [63] did not report the LDL cut-off values they used in their exclusion criteria so it is not possible to assess their reliability [66].

##### Non-Tinnitus Pitch-Matched Stimuli

One study used the audiometric lesion edge (LE) of each tinnitus participant as the tinnitus pitch match [53]. LE was defined as the point just below the frequency at which hearing ability began to deteriorate according to pure-tone audiometric testing. However, a recent study showed that LE is much lower than the tinnitus frequency in people with sensorineural hearing loss, and frequency of maximal hearing loss tended to be just below the tinnitus frequency regardless of the exact configuration of the hearing loss (steep/notched/gradual/inverted “U”) [69]. This homeostatic theory of tinnitus pitch arising from compensatory hyperactivity within the hearing loss area has been supported by other research over the tonotopic model (which posits that the activity occurs in the tonotopic area just below the hearing loss region) (e.g. [45, 46, 70]). Regarding the [53] experiment, the LE frequency was used as a standard tone along with a control standard tone which was one octave below LE. Deviants were 1, 2 or 4% below either of the standard conditions. There was a deviant type dependency in the LE condition, where the 1% deviant elicited a stronger response in the tinnitus group compared to controls while the 2% deviant elicited a weaker response. Furthermore, there was an inverse relationship between tinnitus distress and the 2% deviant MMN amplitudes. Notably, as there was only one other study that looked into *downward* frequency deviants, which used a multi-feature paradigm rather than the classical frequency oddball paradigm, there could be a plethora of reasons for the discrepancy including the differences in sample-matching limitations as well as paradigm contexts (including both presence of other deviant types and far-away frequencies).

Two further studies explored MMN response amplitudes to frequency deviants but did not make an explicit pitch-matching attempt. Both of these used multi-feature paradigms but different frequencies, and one included fewer deviant types than the other. One study, which also included lower frequencies, used frequencies around 5 kHz and found a reduction in MMN amplitudes in response to both *downward* and *upward* frequency deviants in tinnitus [52]. This, however, was the study that had significant differences in age between the two samples, which may have led to the reduction of MMN amplitudes independently of tinnitus (e.g. [47, 57]). Another study investigated potential effects of high tinnitus distress on MMN amplitudes compared to a group with low tinnitus distress and a control group, all of which were hearing and age-matched [71]. The frequencies used in this study were around 8 kHz. All groups showed similar MMN amplitudes in response to *downward* frequency deviants. In response to the *upward* frequency deviants, the low tinnitus distress group had similar MMN amplitudes to controls. However, the high distress tinnitus group had significantly weaker MMN than either of the other two groups. While there is a possibility that the lack of tinnitus pitch-matching also affected this result, it is interesting to compare it with [63], which saw larger MMN amplitudes in the tinnitus group than the controls in response to a deviant tone that was 100 Hz closer in frequency to the tinnitus pitch-match than the standard tones. Both studies matched participants in age and hearing, but [63] excluded participants with suspected hyperacusis by excluding those with low LDLs. LDL scores do sometimes have low sensitivity however, so these tests may miss some cases of the condition [72]. Neither of the tinnitus distress groups in [63] had a similar alteration in response pattern to [71] compared to controls, and the most clear differences between the two comparisons were the paradigm and the explicit lack of hyperacusis (as [71] did not use a hyperacusis measure). However, there is also the possibility that the smaller control group sample size in [63] led to an unrepresentative result.

#### Frequency Deviants: Conclusion

Overall, while a number of studies have explored the effect of tinnitus presence on response to frequency deviation (particularly in lower frequencies), there are still many gaps in the literature. For example, there is a notable lack of downward frequency deviant studies in lower frequencies using classical oddball paradigms, and a lack of differentiation between deviant directionality in multi-feature studies. Furthermore, out of 11 published papers that included a control group and investigated MMN responses to frequency deviants, six did not fully match the groups on hearing and age, and only one explicitly measured and excluded hyperacusis. While the multi-feature paradigm studies have generally been more successful in group matching, these studies did not attempt to look at responses to deviants near the tinnitus pitch (though two authors did use frequencies that possibly were close to tinnitus frequencies [52, 71]). Uniting the positive points from the different studies, such as controlling for hyperacusis, fully matching groups and looking at frequencies both near and away from tinnitus, into one research project may help to clarify discrepancies in the findings so far.

However, allowing for these limitations and caveats, the prevalent finding from most studies has been that MMN response amplitudes are smaller to frequency deviants where the deviant and standard frequencies are distant from the tinnitus frequency.

### Intensity Deviants

Seven studies investigated effects of tinnitus presence on intensity deviant responses. Two of these, both multi-feature paradigm studies, focused only on intensity changes using lower frequency tones distant from the tinnitus frequency [33, 54]. One multi-feature paradigm study utilised both lower and higher frequency tones but did not match the higher tone to tinnitus frequency [52]. Four other studies used a roving oddball paradigm, where a louder tone was pseudo-randomly alternated with a quieter tone and vice versa [35, 43]. Two of these, however, are at the pre-print stage of publication [60, 73]. The quieter tones in these experiments were set at − 6 dB relative to the louder tones.

#### Multi-Feature Paradigm Studies

A study in which the tinnitus group was significantly older on average than the control group showed that there was a reduction in MMN amplitude to both *upward* and *downward* intensity changes both when tones played were around 1 kHz and 5 kHz [52]. These results were supported by [54], which did not differentiate between the two deviant directions but found that overall, tinnitus was associated with reduced MMN amplitude to intensity deviants to tones around 1 kHz. These authors also accounted for cognitive function, including only participants who scored within normal range on the Montreal Cognitive Assessment Test [54]. This is important as some previous research suggested that decreased MMN responses to both upward and downward intensity deviants are related to impaired working memory (e.g. [48, 74]), and decreased MMN responses to *upward* intensity deviants were related to higher age [75], though others found no differences between younger and older participants (with and without depression) or older healthy participants compared to participants of similar age but with mild cognitive impairment [76, 77]. However, another tinnitus study with a similar design to [54] found no differences in MMN amplitudes to intensity deviants in the tinnitus group compared to controls, despite also having hearing and age matched groups [33]. There is a possibility that these differences could be due to varying levels of hyperacusis presence among the different tinnitus samples within these multi-feature studies, because [60] showed that at 1 kHz tones, a group of participants with tinnitus and hyperacusis (T + H +) had weaker responses to intensity deviants than controls, but a group with tinnitus but without hyperacusis (T + H-) showed no such difference.

#### Roving Paradigm Studies

##### Tinnitus-Like Frequencies Only

In one study [7], two near-tinnitus frequencies were used (centre frequency of tinnitus of each participant and edge frequency of tinnitus, which was just noticeably below the tinnitus range of each participant) [43]. Participants with tinnitus had larger MMN responses to *upward* deviants, but smaller MMN responses to *downward* deviants, compared to the control group [43]. During this experiment, and in keeping with most previous studies, a passive task was utilised in which each participant chose a subtitled, silent film to watch during the EEG recording. Another study contrasted results obtained during this passive task with those during an active auditory attention task, and an active visual attention [73]. This study only used the edge frequency of the tinnitus, measured using the method from [43]. The MMN response patterns, both in the tinnitus and the control groups, recorded during the passive task were consistent with [43]. Additionally, the auditory attention task increased the MMN response amplitudes to both upward and downward deviants in the tinnitus group, without qualitatively changing the profile of MMN responses. This was a helpful finding because MMN literature in tinnitus has used both auditory attention tasks and passive attention tasks in the past, and the results of these studies can be meaningfully compared if profiles of response difference between tinnitus and control groups only change quantitatively, rather than qualitatively, on account of this. However, a visual task largely attenuated the MMN responses to *upward* deviants, especially in the tinnitus group compared to controls. In response to *downward* deviants, MMN was present for the tinnitus group, but not for the control group, during the visual task. Importantly, both of the studies described in this section may have been influenced by the presence of hyperacusis in the tinnitus group. In [73], the tinnitus group had an HQ mean score of 16.05, which was both significantly higher than the control group HQ mean score, and just above the suggested cut-off score of 16 [78, 79].

##### Tinnitus-Like and 1 kHz Frequencies

To investigate whether tinnitus is associated with differences in MMN responses to stimulus frequencies unrelated to the region of the hearing loss and tinnitus, a 1-kHz control frequency was used as one of the frequencies within the roving intensity paradigm [35]. The authors also used the edge frequency from [43] as the tinnitus-match frequency because there, the edge frequency showed stronger responses compared to the centre frequency. While results in the tinnitus group were similar to those from the previous study that used very similar (tinnitus centre and tinnitus edge) frequencies, MMN responses to tinnitus frequency tones in the control group became more like those of the tinnitus group once a 1-kHz frequency stimulus condition was added. Thus, these results did not follow the expected pattern in controls and did not show the strength of between-group differences as in the original study [35], for reasons which we explain in the following section.

Notably, none of two aforementioned studies ([35, 43, 73]) controlled for hyperacusis presence, which explains potential discrepancies between the results according to [60] findings. In this study which accounted for hyperacusis, at 1 kHz, the *upward* deviant elicited a significantly weaker MMN response only in the group of T + H + participants while controls and T + H- participants had similar responses. No significant differences, however, were seen between these three groups in response to a *downward* deviant at 1 kHz, though there was a trend (*p* = 0.063) between T + H + and control groups. At the tinnitus frequency, the T + H- group had increased MMN amplitude to *downward* deviants (but similar upward deviant responses to controls), while the T + H + group showed increased MMN responses only to *upward* deviants (but similar downward deviant responses to controls). This finding strongly implies that intensity deviant MMN abnormalities from previous studies are largely reflective of hyperacusis rather than tinnitus per se (with implications for frequency deviants still unknown) and highlights larger MMN to downward intensity deviants in stimuli around the tinnitus frequency as, currently, the sole abnormality specifically attributable to tinnitus.

##### Distance Between Frequencies Affects Intensity Deviant Responses

While [60] could explain some of the differences in tinnitus sample responses, the differences seen in response patterns of the controls remained unexplained. The controls in [60] had similar responses to [35], where the same frequencies were used, but had the opposite pattern to the control group responses in [43]. However, a subsequent study aiming to clarify this discrepancy, which used three healthy control groups (and no tinnitus groups), showed that there was a striking difference in MMN patterns between intensity roving paradigms where different experimental blocks were set at frequencies with a small frequency difference between them (6 kHz and 1/3 octave lower) compared to either a single frequency (6 kHz only) or a larger frequency difference between blocks (6 kHz and 1 kHz) [59]. That is, the presence of different frequency stimuli in remote parts of the same experimental session had a major effect on the MMN response profile to otherwise identical standard and deviant stimuli. The small frequency difference group showed a similar pattern to the control group in [43] (larger MMN to *downward* intensity deviants). Conversely, the single frequency and widely spaced frequencies yielded similar results to [35] and [60], with larger responses to *upward* than downward intensity deviants. There may exist a specific interference effect; in controls, the interference may be due to two nearby alternating stimulus frequencies, whereas in tinnitus, similar interference could have occurred due to alternation between (or simultaneous perception of) the tinnitus itself and the presented tones. It remains to be determined whether the altered pattern of results is an epiphenomenon of tinnitus, a downstream consequence, or whether it might hold insights into key underlying tinnitus mechanisms.

#### Intensity Deviants: Conclusion

In response to stimuli set to around 1 kHz, results seem fairly consistent; differences may stem from the presence of hyperacusis within the tinnitus sample, which reduces the MMN amplitude in comparison to controls. On the other hand, lack of hyperacusis in the tinnitus sample may lead to similar MMN amplitudes between tinnitus and control groups. While an assumption is being made in this conclusion about the three samples in the multi-feature studies, it seems in accordance with the roving paradigm findings [60]. However, the differences seen between T + H + and T + H- might alternatively result from tinnitus distress, rather than hyperacusis, as the two groups had significantly different THI scores (similarly to upward frequency responses in [71]). On the other hand, all three of the multi-feature studies that used lower frequencies [33, 52, 54] had tinnitus samples with higher THI scores than the T + H + group in [60].

In response to roving stimuli at tinnitus-like frequencies, the reported findings also seem consistent, showing both the reproducibility of the findings, and also the implications of different choices of paradigm depending on the type of intensity deviant response abnormality one is trying to highlight [59, 60]. For example, hyperacusis was characterised by increased upward and reduced downward intensity MMNs. The pattern related to hyperacusis might be best revealed using narrowly different frequencies, to give the maximal contrast between the intensity conditions compared to controls. Conversely, tinnitus without hyperacusis was characterised by increased downward intensity deviants, and therefore may be best revealed using single-frequency or widely spaced frequency differences.

### Other Deviant Types

#### Duration Deviants

Overall, six studies included duration deviants. However, this has not been the sole focus of these studies. Within multi-feature paradigms, at frequencies below 5.5 kHz (most often at 1 kHz), tinnitus was associated with reduced MMN amplitudes [33, 52, 54]. However, the studies in which duration deviants were used as a rare control deviant found that the tinnitus and control groups had similar MMN amplitudes to duration deviant in response to both 1 kHz and tinnitus-like frequencies [35, 43]. One potential difference could be the number of included deviant types, as the three studies that found similar responses between the groups had less than four deviant types, whereas the other three studies had at least seven deviant types.

Besides inclusion of a higher frequency, the level of distress experienced by the tinnitus groups may affect the results, as the latter studies had samples with the lower THI mean scores. However, the multi-feature study that used higher (but not necessarily tinnitus-like) frequencies showed no differences between high and low tinnitus distress groups, or between either and the control group [71]. Age difference may explain the findings in [80] as higher age has been linked to lower duration deviant amplitudes (e.g. [55, 81]). Higher levels of depression have been associated with tinnitus, and a review concluded that people with depression or PTSD-related hyper-arousal, as well as healthy people exposed to psychosocial stress-causing cues, tended to show attenuated MMN responses to duration deviants [27, 82, 83]. In adolescents, decreasing MMN responses to duration deviants have been related to increasing general psychological difficulties [84]. Decreased duration deviant amplitude was also associated with extensive negative symptoms and cognitive deficits in people at high risk of psychosis [85].

#### Location Deviants

Location deviants were only studied as part of multi-feature paradigms in three studies [33, 52, 54]. In two of these studies, MMN responses were reduced in the tinnitus group compared to controls [52, 54], but one study did not find a significant difference between the groups [33]. It is not immediately clear why the difference in findings occurred. Similar speculations to differences that caused duration deviant response differences include possible presence of hyperacusis and psychological difficulties that were not measured in the samples. Previous research that explored sound source localisation (but not using MMN) has generally found that tinnitus interferes with this ability especially at a higher frequency [86], but only in certain sound types that included pure tones [87]. However, this may be in part due to effects of hearing loss [88]. Additionally, presence of unilateral tinnitus may increase the localisation difficulties on the same side as tinnitus compared to the opposite side or in cases of bilateral tinnitus [89].

#### Silent Gap (Omission) Deviants

Three multi-feature paradigm studies found decreased MMN responses to silent gap deviants compared to controls [33, 52, 54], while one found that only the high distress tinnitus group had reduced MMN, but not the low distress tinnitus group [71]. The low distress group had much lower THI scores than any other group in these four studies, so it is possible that that tinnitus severity is related to the reduced silent gap deviant MMN response. Alternatively, the higher frequencies used in [71] compared to the other studies may have been related to the differences in a way that has not yet been explored. Frequencies above 4 kHz were also used in [52] but due to the inclusion of 1 kHz blocks and the differences in age of the groups, the reason for their result was unclear [90].

A review (including studies that did not use MMN measures) concluded that there is a possibility that tinnitus does cause participants to require a longer gap to detect it compared to controls, as this was found in seven studies that used various types of gap detection tests [91]. An additional important factor may have been musical education in the two groups, as gap detection tests scores were significantly different between musicians and non-musicians [92].

#### Other Deviant Types: Conclusion

Overall, to understand whether tinnitus presence has an impact on detection of duration, location or silent gap deviants, more research is needed that focuses specifically on these types of deviants. The duration, silent gap and location deviants may reflect psychological/cognitive factors that may or may not stem from tinnitus, rather than the percept itself. However, this conclusion is based on speculation from previous literature. Location deviant responses may also reflect auditory factors such as hearing loss or tinnitus lateralisation. Silent gap detection may be related to tinnitus presence, but this conclusion is also not based on MMN literature, and they may also reflect musical experience.

## Discussion

Overall, the simple sound features that have been most researched and are likely the most relevant to the tinnitus percept rather than related symptoms are frequency and intensity. Frequency deviants have been studied for longer, but require more tightly controlled samples in terms of hearing and age, as currently the true mechanisms behind the differences seen in tinnitus groups compared to controls remain inconclusive. Additionally, splitting directionality of the deviants, and studying both upward and downward frequency deviants, is needed to understand how widespread the association between tinnitus and MMN alterations is. Intensity deviant findings still need to be replicated to determine whether these can allow researchers to differentiate between tinnitus with and without hyperacusis. Another useful direction would be to understand whether the findings in all deviant types could be partially explained by tinnitus distress rather than hyperacusis.

Groups with tinnitus and without hyperacusis group reflect the closest thing to a “pure tinnitus” measure obtained within the intensity oddball paradigms, possibly similarly to [63] within frequency oddball paradigms. However, measures by which the presence of hyperacusis was determined differed, with one study using LDLs and the other using HQ and IHS scores. Some inconsistencies between LDLs and questionnaire scores have been previously found, especially with tinnitus also present, and it has been suggested that both measures should be used to identify hyperacusis due to differing specificity and sensitivity of the two measures [72]. Essentially, LDL scores have high specificity, and if LDLs show altered values, one can be confident that participants have hyperacusis; however, these tests may miss some cases of the condition. HQ scores have high sensitivity, which would allow to correctly include participants with hyperacusis as they do not miss true cases, but the scores may be altered even in participants without hyperacusis. Therefore, [63] may have erroneously kept some participants with hyperacusis, while in [60] there may be some erroneously included participants within the T + H + group that did not truly have hyperacusis. However, this would only have weakened the findings, yet the findings for T + H + were strong. IHS scores were also collected to determine presence of hyperacusis in [60], but this measure may also reflect tinnitus distress, anxiety and depression, which may reduce its selectivity for hyperacusis as opposed to tinnitus [93]. In the future, an interesting self-report to use would be the Hyperacusis Assessment Questionnaire, which has been reported to measure subcategories of hyperacusis without the limitations found in IHS [94].

It may also be interesting to understand effects of certain emotions and cues on change detection in people with tinnitus. In some mentioned experiments, participants watched silent films during EEG recordings (e.g. [35]). However, it has been found that seeing positive pictures was related to larger MMN amplitudes to intensity and duration deviants, compared to negative or neutral pictures [95]. Auditory emotional cues (happy versus sad music) can also alter responses; visual cues could alter responses even to standard tones when a misleading cue had been presented beforehand [96, 97]. Aversive and more arousing sound elicited larger MMN amplitudes in healthy participants [98]. Studying differences in cue and emotion-based auditory predictions, and their violations, could add a whole additional layer of understanding to our knowledge about differences in sensory processing associated with tinnitus, and mechanisms of the percept itself compared to effects of tinnitus-related distress.

A potential limitation of all mentioned MMN research is that the studies were not longitudinal. It may help to understand how the differences in sound processing develop over time in intermittent tinnitus and during the acute stage of constant tinnitus, as this could shine light on the development and divergence of the alterations in different mechanisms related to tinnitus/combination of tinnitus and comorbid conditions. It would be useful to understand whether MMN changes occur before, or follow after, tinnitus development.

Another important factor may be whether or not participants had undergone any tinnitus treatment. While many studies recruited from ENT and tinnitus clinics, a large proportion did not report whether any treatment was received prior to the experiment [42, 50, 51, 53, 65]. Other studies, which recruited via non-clinical routes, also did not report whether participants with tinnitus had ever undergone tinnitus treatment [35, 43, 59, 60, 73]. However, it may be important to understand how treatment (or tinnitus being non-bothersome) may alter MMN amplitudes, because two studies, in which two different types of tinnitus treatment were used, showed that MMN amplitudes in response to frequency deviants in participants with tinnitus became more similar to MMN amplitudes in controls, compared to MMN amplitudes seen pre-treatment [49, 63]. This pattern should be studied in order to understand whether MMN amplitudes can indicate tinnitus presence overall, or only presence of bothersome tinnitus.

Within the limitations and caveats of this field of research, we can say that (1) the presence of tinnitus and/or hyperacusis seems to be fairly consistently associated with reduced MMN amplitude to frequency deviants (and seemingly other types of deviant) where stimuli are remote from the tinnitus frequency, but it is unclear how, and how directly, this relates to core mechanisms of tinnitus itself; (2) the presence of hyperacusis, with or without tinnitus, is associated with increased MMN amplitude to upward intensity deviants at the affected frequencies; (3) tinnitus without hyperacusis may be associated with increased MMN amplitude to downward intensity deviants around the tinnitus frequency, with effects on frequency deviants unknown; and (4) many factors peripherally related to tinnitus quantitatively affect the amplitude of MMN responses, including attention/task, and the overall range of stimuli used in the experiment.

To conclude, MMN studies may provide interesting and useful insight into tinnitus mechanisms; however, more well-controlled investigations are still needed to fully grasp the meaning behind MMN alterations associated with tinnitus. It would be beneficial to include tight hearing and age-matching, questionnaires about mental health/cognitive ability and LDLs and self-reports about hyperacusis.

## Supplementary Information

Below is the link to the electronic supplementary material.

ESM 1 (PNG 891 KB)

High Resolution Image (TIFF 10.7 MB)
