# Supplementary figures and images for: What Do Mismatch Negativity (MMN) Responses Tell Us About Tinnitus?
Source: J Assoc Res Otolaryngol. 2024 Dec 16;26(1):33–47. doi: 10.1007/s10162-024-00970-1 (PMC11861849; doi:10.1007/s10162-024-00970-1)

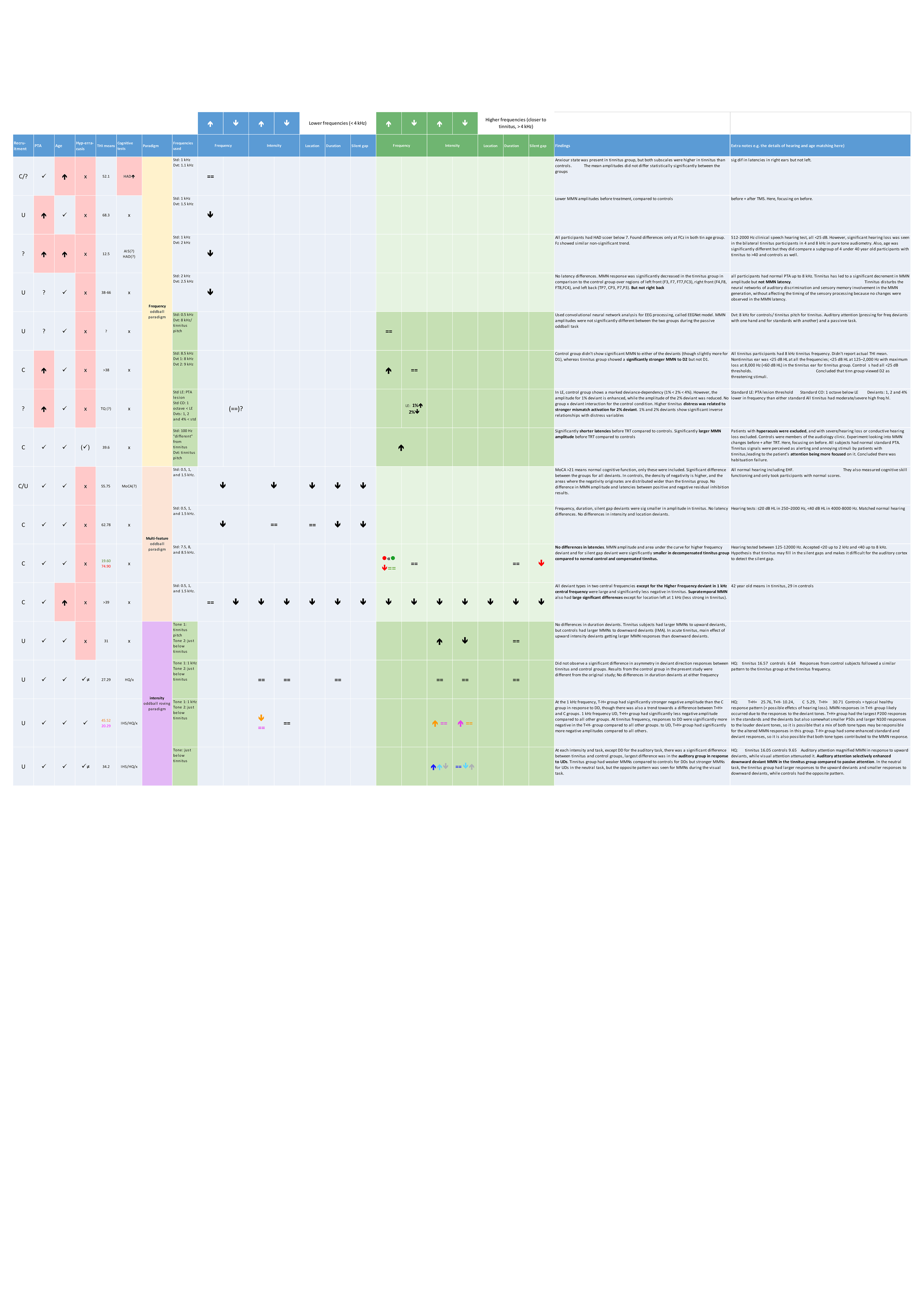

Supplement: Supplementary file 1 — (PNG 891 KB) [file 10162_2024_970_Fig6_ESM.png]
